# Secondary language impairment in posterior cortical atrophy: insights from sentence repetition

**DOI:** 10.3389/fnins.2024.1359186

**Published:** 2024-03-21

**Authors:** Samrah Ahmed, Josie Caswell, Christopher R. Butler, Arpita Bose

**Affiliations:** ^1^School of Psychology and Clinical Language Sciences, University of Reading, Reading, United Kingdom; ^2^Faculty of Medicine, Department of Brain Sciences, Imperial College London, London, United Kingdom

**Keywords:** posterior cortical atrophy, Alzheimer’s disease, sentence repetition, language impairment, phonology

## Abstract

**Introduction:**

Posterior cortical atrophy (PCA) is a neurodegenerative syndrome characterized by progressive impairment in visuospatial and perceptual function linked to atrophy of the occipito-parietal cortex. Besides the salient visual impairment, several studies have documented subtle changes in language may also be present. Sentence repetition is a highly constrained linguistic task involving multiple linguistic and cognitive processes and have been shown to be impaired in other AD spectrum disorders, with little consensus on its relevance in PCA. This aim of this study was to further delineate the linguistic and cognitive features of impaired language in PCA using a sentence repetition task.

**Method:**

Seven PCA patients and 16 healthy controls verbally repeated 16 sentences from the Boston Diagnostic Aphasia Examination. Responses were transcribed orthographically and coded for accuracy (percentage accuracy; percentage Correct Information Units; Levenshtein Distance) and for temporal characteristics (preparation duration (ms); utterance duration (ms); silent pause duration (ms); speech duration (ms); dysfluency duration (ms)). The potential modulating effects of attentional control and working memory capacity were explored.

**Results:**

PCA patients showed lower overall accuracy with retained semantic content of the sentences, and lower phonological accuracy. Temporal measures revealed longer preparation and utterance duration for PCA patients compared to controls, alongside longer speech duration but comparable dysfluency duration. PCA patients also showed comparable silent pause duration to controls. Attentional control, measured using the Hayling sentence completion task, predicted accuracy of sentence repetition.

**Discussion:**

The findings suggest that sentence repetition is impaired in PCA and is characterized by phonological, response planning and execution difficulties, underpinned in part by attentional control mechanisms. The emerging profile of language impairment in PCA suggests vulnerability of similar cognitive systems to other Alzheimer’s syndromes, with subtle differences in clinical presentation.

## 1 Introduction

Posterior cortical atrophy (PCA) is a dementia syndrome characterized by progressive visual impairment. Patients have difficulties with recognizing faces and objects, and navigating their surroundings (Tang-Wai et al., 2004). The most common underlying cause is Alzheimer’s pathology (Renner et al., 2004), and prevalence studies suggest as many as 15% of people diagnosed with Alzheimer’s disease (AD) could have PCA (Hendriks et al., 2021).

Besides the salient visual impairment, several studies have shown that some PCA patients also develop subtle and progressive language difficulties (Migliaccio et al., 2009; Crutch et al., 2017). The earliest descriptions of PCA describe language disturbances as a transcortical sensory aphasia (Benson et al., 1988) characterized by anomia and mild changes in speech comprehension (Freedman et al., 1991). More recent research has revealed a “logopenic” profile similar to, but much milder, than the language deficits observed in logopenic variant primary progressive aphasia (LvPPA), comprising symptoms associated with parietal and posterior temporal brain regions that characterize the degeneration in PCA. Like lvPPA, PCA patients have difficulty with auditory input processing, measured by impaired perception of linguistic prosody (Crutch et al., 2013), slowed speech rate and anomia (Crutch et al., 2013; Magnin et al., 2013; Mitchell et al., 2017) and phonological errors on naming tasks (Gorno-Tempini et al., 2008; Tetzloff et al., 2021).

Consistent with the core criterion for impaired sentence repetition in lvPPA (Gorno-Tempini et al., 2008, 2011), PCA patients have also been reported to have difficulty with sentence repetition (Crutch et al., 2013; Magnin et al., 2013). However, a closer examination of sentence repetition findings in PCA suggest the true relevance of this particular impairment remains unclear. Sentence repetition in PCA has thus far only been examined to determine the presence or absence of a deficit, with no detailed characterization of performance. Crutch et al. (2013) utilized ten sentences of 4–5 words in a cohort of 15 PCA patients. The authors reported poor repetition performance in their PCA group. Examination of individual performance revealed that, in fact, where 100% of LPA patients performed below the 10th centile, only 47% of PCA patients performed at this level, and only 2/15 scored less than 90%. Similarly, Magnin et al. (2013) used sentence repetition of three items in a cohort of 9 PCA patients. 55% of PCA patients exhibited a repetition deficit. It remains unclear whether impaired sentence repetition is a ubiquitous feature in the PCA language profile, or if impairment reflects inherent heterogeneity within the patient sample. Furthermore, both studies reported little beyond accuracy judgments, or the nature of cognitive mechanisms modulating performance.

Sentence repetition requires immediate verbal repetition of orally-presented sentences. The ability to accurately repeat verbal information involves multiple linguistic and cognitive processes and has traditionally been linked to auditory verbal short term memory and lexical retrieval, critically mediated by cortical regions in left posterior temporo-parietal cortex (Paulesu et al., 1993). Accurate sentence repetition requires phonemic integration of sounds in order to form each individual word (DeLeon et al., 2007), temporary storage of these items maintained by verbal short term memory (Baddeley, 2003), and the capacity to effectively bind smaller items into chunks of meaningful information to allow for accurate retention (Baddeley et al., 2009). Experimental observations suggest that, whilst a phonological trace is still held in short term memory, the actual retrieval of the words for repetition is achieved by a process of reconstruction at a conceptual level, originating in long-term memory and mediated via attentional control (Dell et al., 2007; Riches et al., 2010; Riches, 2012). Sentence repetition further necessitates multiple linguistic processes. Accurately recalling a sentence involves parsing the sentence, analyzing the thematic relations (i.e., the order of events), interpreting the underlying syntactic representation, elaborating an articulation plan and, finally, producing the sentence (Rujas et al., 2021).

Impaired sentence repetition is a common feature in AD spectrum disorders. LvPPA have striking impairment in sentence repetition, consistently linked to impairment in the phonological loop of auditory short term memory, and associated with atrophy in the left posterior temporo-parietal region (Gorno-Tempini et al., 2004, 2008; Leyton et al., 2014). Although amnestic AD is not characterized by severe language impairment, secondary impairment in sentence repetition has been well documented, similarly associated with reduced capacity for working memory resources (Perry and Hodges, 1999; Peters et al., 2007) and lexical semantic retrieval (Hodges et al., 1992; Peters et al., 2009). Given what we know of the overlapping cognitive and anatomical changes in PCA, it is likely that sentence repetition is also of greater relevance in the language profile of PCA than previously documented. PCA patients show poor lexical phonological controlled retrieval (e.g., on letter fluency tasks) and poor lexical semantic retrieval (e.g., impaired naming, category fluency and word finding in spontaneous speech) (Crutch et al., 2013; Magnin et al., 2013; Mitchell et al., 2017). Furthermore, encoding and retrieval are selectively impaired in PCA, secondary to working memory, attentional and lexical retrieval deficits arising from lateral parietal atrophy (Ahmed et al., 2018) and frontoparietal network disruption (Putcha et al., 2018; Veldsman et al., 2019). These cognitive mechanisms have the capacity to modulate sentence repetition performance in PCA.

The primary aim of this study was to profile the nature of sentence repetition performance in PCA at greater depth, beyond accuracy judgments. Given that PCA patients are suspected to exhibit subtle language deficits, more nuanced performance measures were used. These captured accuracy of sentence repetition related to semantic and phonological processing, and temporal characteristics that captured different stages of sentence repetition performance, ranging from time taken to start the production of the sentence, pauses between words, and duration of the speech segments, among others. These measures allow precise quantification of an individuals’ responses, and are useful for isolating the specific mechanisms or stages in sentence repetition that may highlight subtle impairment in PCA. Temporal measures can detect language difficulties not picked up by accuracy scores, as evident from the latent aphasia literature (Fromm et al., 2017; DeDe and Salis, 2020; Salis et al., 2021).

Based on the literature to date, we hypothesized that PCA patients would perform more poorly on sentence repetition tasks compared to healthy controls. More specifically, we predicted that PCA patients would show reduced accuracy and impaired temporal characteristics of sentence repetition, related to semantic and phonological aspects of production. Secondly, we hypothesized that impaired sentence repetition would be modulated by attentional control and working memory capacity.

## 2 Materials and methods

### 2.1 Participants

Seven PCA patients were recruited through the Oxford Cognitive Disorders Clinic, Oxford, UK. Diagnosis was established by a senior behavioral neurologist and neuropsychologist. All patients fulfilled consensus criteria for PCA (Tang-Wai et al., 2004; Crutch et al., 2017), based upon clinical assessment, brain imaging and detailed neuropsychological assessment. Patients showed marked impairment in visuospatial and visuoperceptual skills, with relatively preserved behavior and personality. Clinical magnetic resonance imaging (MRI) confirmed characteristic focal atrophy in the occipital and parietal lobes consistent with a diagnosis of PCA. Sixteen control participants were recruited from the local Oxfordshire community. Control participants had no objective cognitive impairment [scored >88 on the Addenbrooke’s Cognitive Examination II (ACE-III) (Hsieh et al., 2013)], and no prior history of psychiatric illness or significant head injury, and were not prescribed any medication known to affect cognition. All participants reported no hearing loss that impaired everyday communication and interaction. PCA patients and controls were matched for age, and years of education. PCA patients experienced symptoms for an average of 3.5 years (SD 1.1 years) (Table 1). The study was approved by the National Research Ethics Service South Central - Hampshire B and Oxford C. All participants provided written informed consent in accordance with the Declaration of Helsinki.

**Table 1:** Demographic and clinical characteristics of control and PCA groups. Standard deviation given in parentheses. Total scores achievable on neuropsychological tests, where applicable, in parentheses in right column. Values in bold indicate significant group differences.

	Controls (*n* = 16)	PCA (*n* = 7)	*p*
Demographics			
Age (yrs)	62.6 (6.3)	66.1 (5.6)	0.209
Education (yrs)	17.1 (3.3)	16.4 (2.6)	0.719
Sex (male:female)	8:8	2:5	-
Handedness (left: right)	1:15	0:6	-
Race	White Caucasian	White Caucasian	-
Symptom duration (yrs)	-	3.5 (1.1)	-
Background neuropsychology			
ACE-III (100)	97.0 (2.7)	67.1 (12.4)	**<0.001**
ACE-III attention (18)	17.2 (1.6)	13.1 (2.3)	**<0.001**
ACE-III memory (26)	25.1 (1.4)	17.9 (5.1)	**<0.001**
ACE-III fluency (14)	13.1 (1.4)	8.4 (3.4)	**<0.001**
ACE-III language (26)	25.6 (0.81)	21.9 (3.5)	**<0.001**
ACE-III visuospatial (16)	15.9 (0.25)	5.9 (3.7)	**<0.001**
VOSP dot counting (10)	9.9 (0.34)	6.0 (3.1)^†^	**0.002**
VOSP position discrimination (20)	19.6 (0.63)	11.2 (1.9)^†^	**<0.001**
VOSP cube analysis (10)	9.7 (0.48)	1.3 (1.4)^†^	**<0.001**
Rey-Osterrrieth Complex figure copy (18)	18.0 (0)	3.9 (3.7)	**<0.001**
Rey-Osterrrieth Complex figure immediate recall (18)	13.6 (2.9)	0.57 (0.98)	**<0.001**
Rey-Osterrrieth Complex figure delayed recall (18)	13.6 (3.1)	0.14 (0.38)	**<0.001**
PPT (52)	51.6 (0.63)	48.9 (2.5)	**0.022**
Category fluency	24.3 (4.5)	12.1 (4.1)	**<0.001**
FAS letter fluency	50.4 (10.3)	29.0 (15.6)^†^	**0.002**
Boston word repetition (10)	9.94 (0.25)	10 (0)	0.508
FCSRT encoding (16)	16.0 (0)	15.7 (0.82)^†^	0.590
FCSRT total free recall (48)	34.3 (4.6)	16.8 (15.0)^†^	**0.010**
FCSRT total cued recall (48)	13.7 (4.5)	18.3 (12.2)^†^	0.400
FCSRT total recall (48)	47.9 (0.25)	35.2 (16.4)^†^	**0.002**
Working memory			
Digit span forwards (16)	10.1 (1.3)	10.4 (2.2)	0.628
Digit span backwards (16)	8.8 (2.8)	5.7 (0.52)^†^	**0.002**
Digit span total (32)	18.9 (3.6)	16.5 (2.0)^†^	0.294
Attentional control			
Hayling Section 1 response initiation	4.4 (0.81)	3.0 (0)	**0.001**
Hayling Section 2 response inhibition	5.8 (0.68)	3.9 (1.5)	**0.002**
Hayling Section 2 errors	6.4 (1.0)	5.1 (2.2)	0.198
Hayling Overall score	5.5 (0.82)	3.1 (1.8)	**<0.001**

PCA, Posterior cortical atrophy; ACE, Addenbrooke’s Cognitive Examination; VOSP, Visual object and space perception; RAVLT, Rey auditory verbal learning task; PPT, Pyramids and Palm Trees; FCSRT, Free and cued selective reminding test. ^†^Missing data: Data was missing for one PCA patient, who was unable to complete the task.

### 2.2 Neuropsychological profile

Standardized neuropsychological tests were administered to evaluate patient and control participant function in four domains:

- *Global cognition*: Addenbrooke’s Cognitive Examination-III [ACE III; (Hsieh et al., 2013)] consisting of assessment of attention, memory, fluency, language and visuospatial skill.
- *Visuospatial function*: Dot counting, position discrimination and cube analysis from the Visual Object and Space Perception [VOSP; (Warrington and James, 1991)] and the Rey-Osterrieth Complex figure (Rey, 1941).
- *Language*: Oral Pyramids and Palm Trees [PPT; (Howard and Patterson, 1992)], category fluency (Morris et al., 1989), FAS letter fluency (Benton and Hamsher, 1976) and Boston word repetition (Goodglass and Kaplan, 1972).
- *Episodic memory*: Free and Cued Selective Reminding test [FCSRT; (Grober et al., 2000)].

### 2.3 Sentence repetition stimuli and procedure

Sixteen sentences from the Boston Diagnostic Aphasia Examination (Goodglass and Kaplan, 1972) were used as stimulus material (Table 2). The sentences ranged from 3–11 syllables. Sentences were presented in the same fixed order to all participants. Participants were asked to repeat each of the sentences after the examiner, one at a time. Repeat presentation of the stimulus and self-corrections were allowed, but the response was recorded as incorrect. Only responses following the initial presentation of a sentence were included in the analyses. Both the examiner’s and participant’s productions were audio recorded and saved as m4a or mp3 files. The recordings were transcribed for further analysis.

**Table 2:** Examples of PCA performance on sentence repetition from the Boston Diagnostic Aphasia Examination (Goodglass and Kaplan, 1972).

	Stimulus	Examples from various PCA patients	Accuracy (1 correct; 0 incorrect)	CIU%	LD
1	You know how	You know how	1	100	0
2	The vat leaks	The fat leaks	0	100	1
3	Down to earth	Down to earth	1	100	0
4	Limes are sour	Lines are sour	0	67	1
5	I got home from work	I got home from church	0	100	1
6	The spy fled to Greece	The spy .sl. fled to Greece	0	100	1
7	You should not tell her	You should not tell her	1	100	0
8	Pry the tin lid off	Prise the tin lid off	0	80	1
9	Go ahead and do it if possible	Go ahead and do it if possible	1	100	0
10	The Chinese fan had a rare emerald	The Chinese fan had a rare emerald	1	100	0
11	Near the table in the dining room	Near the table in the dining room	1	100	0
12	The barn swallow captured a plump worm	The barn s-swallow captured a worm	0	86	2
13	They heard him speak on the radio last night	They heard him speak on the radio last night	1	100	0
14	The lawyer’s closing argument convinced him	The lawyer’s closing argument… convinced him	1	100	0
15	I stopped at his front door and rang the bell	I stopped at his front. Door and rang the bell	1	100	0
16	The phantom soared across the foggy heath	The phantom soared across the… foggy heath	1	100	0

CIU, Correct Information Unit; LD, Levenshtein Distance.

### 2.4 Data extraction and analysis

Data extraction and coding was undertaken by JC and AB. Participants’ responses were transcribed orthographically, except where phonological, phonetic or neologistic errors were made, in which case the international phonetic alphabet was used. Any discrepancies in transcription and error coding were discussed and resolved in consultation. The following measures were extracted and coded (see Table 2 for examples):

- Accuracy

*Percentage accuracy*: Defined as the percentage of accurate responses. Any response deviating in any way from the original stimulus was considered incorrect. Requests to repeat the stimulus resulted in a coding of incorrect.

*Percentage Correct Information Units (CIU%)*: CIU captures the proportion of superfluous words that are not related to the stimuli that participants may insert when attempting to repeat, reflecting their difficulty in maintaining the semantic content of the stimuli. CIUs have been extensively used in spontaneous speech analysis in various neurological populations, including, post-stroke aphasia and AD to measure semantic informativeness and meaningfulness of production (Nicholas and Brookshire, 1993; Ahmed et al., 2013; Bose et al., 2021). It is calculated by dividing the total number of correct information units (words that are intelligible in context, relevant to, informative about and accurate in relation to the stimulus) by total number of words in the response. Only complete and recognizable words were included in the count.

*Levenshtein Distance (LD):* Levenshtein distance is a lexical similarity measure which identifies the distance between the target and the response, and represents the smallest number of changes required to transform a recorded response to the original stimulus. It does so by counting the number of times one would have to insert, delete or substitute a character from response to target. For LD calculation, an algorithm using an Excel macro was used to arrive at the minimum number of changes (including substitutions, omissions, additions) between stimulus and response. For scores greater than zero (i.e., where errors occurred), a manual check of LD was made to confirm correct calculation. Unlike CIUs, filled pauses and false starts were included in the calculation. To a large extent, LD captures the phonological changes in the repetition of the target sentences. In the pediatric (Riches et al., 2010; Riches, 2012) and aphasia literature (Salis et al., 2021), LD has been shown to have better diagnostic accuracy than simple accuracy measures.

- Temporal characteristics

Five measures were adapted from Salis et al. (2021):

*Preparation duration (ms):* Duration of the silent period at the end of the examiner’s spoken stimulus sentence and initiation of the participants’ spoken response. It is thought to reflect lexical and semantic processing and sentence planning but not the actual articulation of the speech segments (Salis et al., 2018; DeDe and Salis, 2020).

*Utterance duration (ms):* The time from initiation of the participant’s response to the end of their spoken output, capturing ongoing lexical, semantic, sentence planning processes, pauses and the articulation of the speech segments. It included spoken words, silent pauses (pauses between words), mazes (non-propositional content, e.g., “I do not know”; “I cannot remember”) and dysfluencies (e.g., false starts, repetitions, repairs) within the response. However, silent pauses or mazes that appeared at the end of a response were not included as they were considered indications that the response attempt was finished. Dysfluencies included false starts, repairs, repetitions and filled pauses.

*Silent pause duration (ms):* Duration of all silent pauses longer than 150 ms within a participant’s response. Pauses greater than 200 ms are thought to reflect dysfluencies in neurotypical adults (Mack et al., 2015), but were considered to be insufficiently sensitive to detect nuanced performance differences (Salis et al., 2018), so the threshold was lowered to 150 ms. Silent pauses within a response are thought to reflect lexical selection, retrieval, and monitoring and have been proposed to tap into word-finding difficulties (Salis et al., 2018).

*Speech duration (ms):* Duration of the speech segments, excluding any pauses (e.g., silent or filled pauses, false starts, repaired sequences, repetitions or mazes) within the response. This measure captures the speed of articulation and reflects motor speech performance.

*Dysfluency duration (ms):* Dysfluency duration includes the combined duration of different types of disfluencies (e.g., filled pauses within a response, false starts, repetition, repaired sequences, mazes).

**Figure 1:**
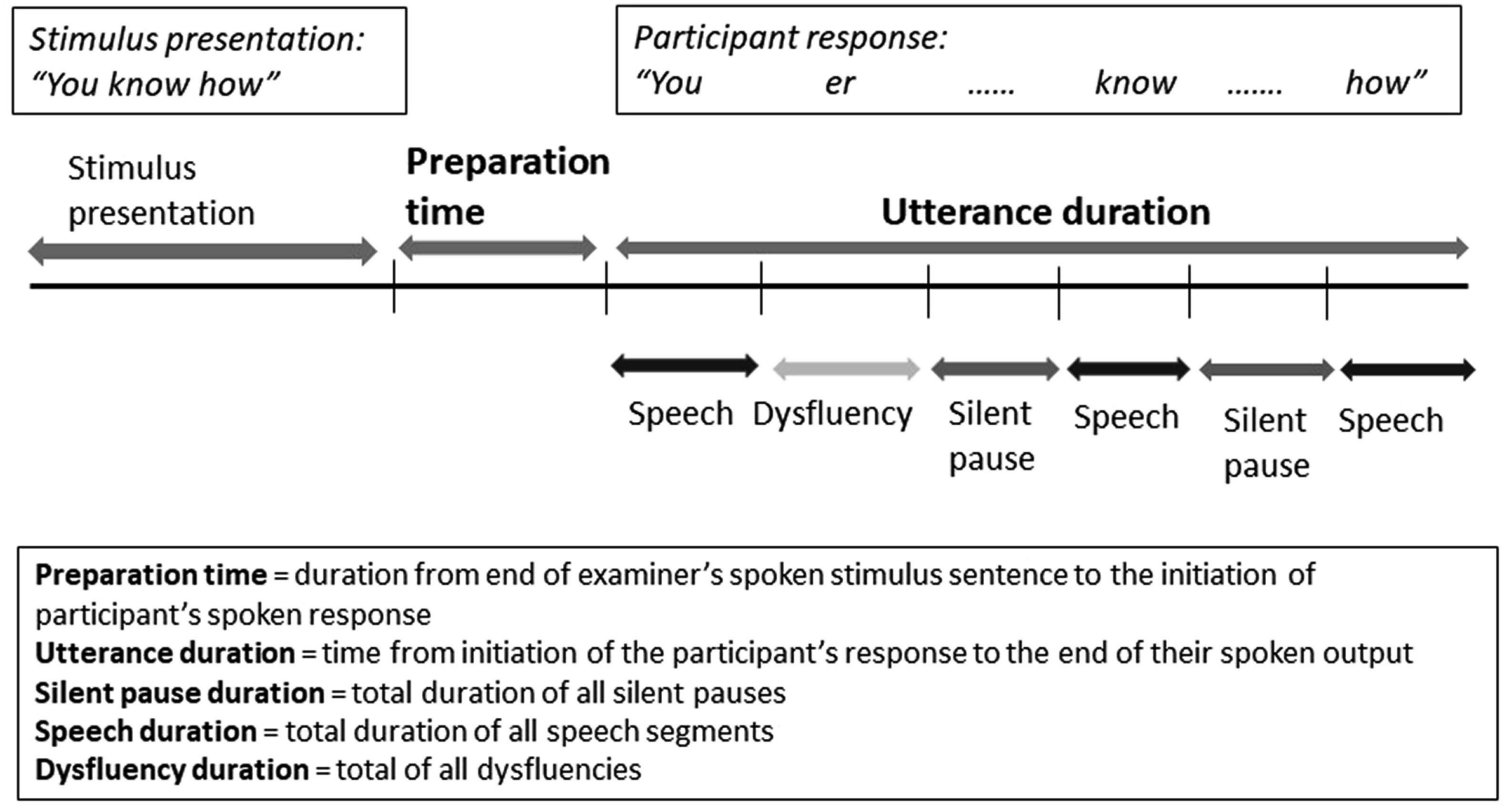
Illustration of segmentation of participants’ responses into temporal measures.

For the temporal analyses, sound files were imported into the speech analysis software PRAAT, which produced spectrographs of all recordings. With minimum silent pause duration set at 150 ms, the software segmented the recordings into silent pauses and speech. These segments were then adjusted by hand. Orthographic transcriptions were added to identify participant and experimenter output. The PRAAT output was then imported into Excel for analysis. See Figure 1 for illustration of segmentation process for each response.

### 2.5 Working memory and attentional control assessment

Working memory was defined here as the limited processing resource that enables online maintenance of verbal information and its executive control (Miller et al., 2018). Working memory was assessed using Digit span forwards and backwards (Weschler, 2014). Participants were verbally presented a series of numbers to repeat, either forwards or in backward order. In the forward test, participants began by being asked to repeat a sequence of three numbers verbatim. Participants continued to repeat incrementally longer sequences to a maximum of 9 number sequences. Participants were given two trials to hear and repeat each of the sequences presented, and testing was ended if errors were made on two consecutive trials. One point was awarded for each correctly repeated trial. The same procedure was followed for digit span backwards beginning with 2 numbers to be repeated backwards, to a maximum of 8 numbers. Since each task places different demands on working memory capacity, data analysis explored the effect of three variables: digit span forwards, backwards and digit span combined. Storage and computational demands are least in digit span forwards, and greatest in digit span backwards where additional processing is needed to manipulate the sequence of numbers in reverse order.

Attentional control was measured using the Hayling Sentence Completion Task (Burgess and Shallice, 1996) which assesses both response initiation (Section 1) and response inhibition (Section 2). The test consists of two sets of 15 sentences, each with the last word missing. In Section 1, the examiner read each sentence aloud and the participant was asked to complete the sentence by generating an appropriate word, providing a measure of response initiation accuracy and speed (measure by a stopwatch). In Section 2, the participant was asked to complete the sentence by generating a word that did not correctly complete the sentence and was not connected to the sentence, giving a measure of response inhibition ability and speed. For Section 1 and 2, response latencies were recorded in whole second units and converted to scaled scores. Section 2 error score was calculated by coding the responses into one of 3 categories: direct sentence completion (category A), somewhat related (category B) or unrelated. Category A and B errors were summed and transformed to a scaled score. The sum of the three scaled scores was converted to an overall score.

### 2.6 Statistical analysis

Demographic, neuropsychological and experimental data were explored using independent sample t-tests or Mann–Whitney tests for non-parametric indices, as appropriate. Alpha level was set at 0.05. Multiple linear regression analyses were conducted to explore the association between working memory and attentional control, and three linguistic measures: (1) accuracy; (2) LD as an indication of phonological processing; and (3) speech duration as a measure of articulatory and phonological processes.

## 3 Results

### 3.1 Neuropsychological profile

PCA patients were significantly impaired on all visuospatial and perceptual tasks, in keeping with the salient profile of impairment in PCA. Tests of expressive language and fluency were also impaired. PCA patients showed some impairment in episodic memory – encoding of a list of words on the FCSRT was similar to controls but free recall after a short delay was impaired. Patients could be cued to recall the missing words, to bring their recall in line with controls (see Table 1).

### 3.2 Sentence repetition performance

- Accuracy

**Figure 2:**
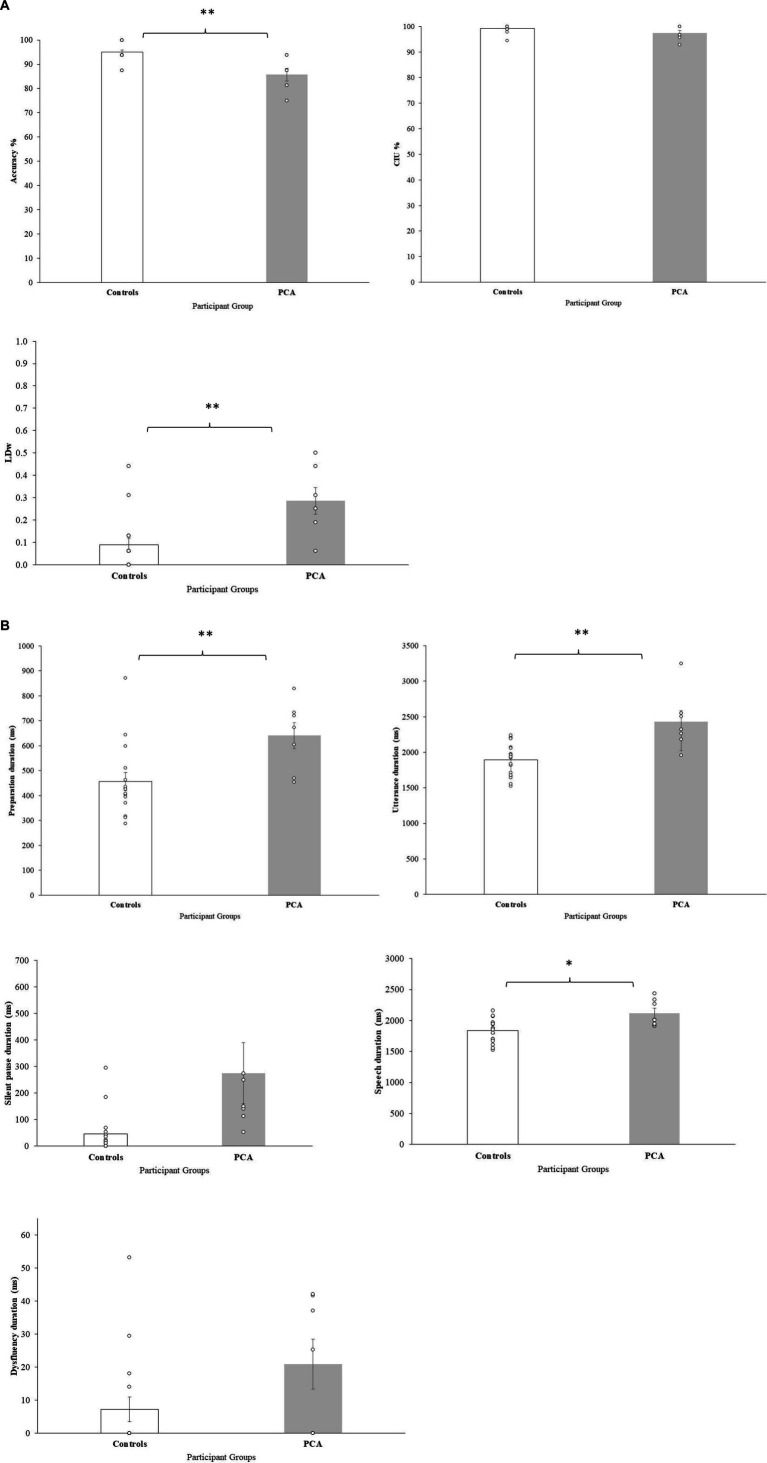
Sentence repetition variables **(A)** accuracy measures, **(B)** temporal measures. Bars represent group mean, errors bars represent standard errors. Individual participants represented by dots.

PCA patients generated significantly more errors than controls, with lower overall accuracy scores. CIU%, capturing semantic informativeness and content, remained comparable to the controls, with PCA patients performing almost at ceiling. In contrast, PCA patients showed significantly higher LD indicating poorer performance, and reflecting difficulties with phonological processing with increased use of substitutions, omissions, and additions of sounds (Figure 2A). Taken together, these findings indicate that CIU and LD captured two different aspects of accuracy, and suggest that for PCA patients the difficulty in accuracy could be originating at the phonological level (see examples in Table 2).

- Temporal characteristics

PCA patients showed significantly longer preparation duration and longer utterance duration. Longer preparation duration is a somewhat predictable finding, given that most neurological conditions commonly present with slower processing resulting in lengthening of duration to initiate a response. However, increased utterance duration could be due to various reasons: increased silent pause duration, increased speech duration or increased time for dysfluencies. PCA patients and controls showed no significant difference in silent pauses but patients showed increased speech duration, suggesting that PCA patients took significantly longer to articulate the words of the sentences. With regards to dysfluency duration, there was no significant difference between groups. Dysfluencies included various categories (i.e., filled pauses, false starts, repaired sequences, repetition, and mazes), however only two categories of dysfluencies (i.e., false starts and repaired sequences) were evident in data from PCA patients. Taken together, longer speech duration but comparable dysfluency duration suggest that PCA patients might not have a generalized motor speech issue, but have specific difficulties when they are required to articulate meaningful lexical items (Table 3; Figure 2B).

**Table 3:** Performance on sentence repetition variables in PCA and control groups. Means, with standard deviations in parentheses. Values in bold indicate significant group differences.

	Controls (*n* = 16)	PCA (*n* = 7)	*p*	z-score	Effect size	Interpretation in PCA group
Accuracy measures						
Accuracy %	95.0 (4.1)	85.7 (7.0)	0.003	−2.927	−0.56	Lower accuracy
CIU %	99.2 (1.45)	97.4 (2.75)	0.139	−1.48	−0.28	Similar to controls
LD*	0.09 (0.12)	0.29 (0.15)	0.005	2.781	0.54	Higher number of errors
Temporal measures*						
Preparation duration (ms)	456.0 (145.6)	640.4 (139.6)	0.006	2.739	0.53	Longer preparation time
Utterance duration (ms)	1896.5 (228.7)	2432.5 (409.2)	0.002	3.074	0.59	Longer utterance time
Silent pause duration (ms)	46.3 (80.9)	274.9 (305.2)	0.447	1.443	0.28	Similar to controls
Speech duration (ms)	1835.9 (193.6)	2118.2 (220.4)	0.034	2.683	0.52	Longer speech time
Dysfluency duration (ms)	7.2 (15.03)	20.7 (20.3)	0.143	1.26	0.18	Similar to controls

*Higher scores on LDw and temporal measures indicates poorer performance. CIU, Correct Information Unit; LD, Levenshtein Distance.

In summary, a fine-grained approach to analyze sentence repetition performance using selected accuracy and temporal measures highlights that difficulty in sentence repetition is apparent in PCA, and difficulties could be mediated by difficulty in planning of the sentences, phonological processing, as well as in the actual articulation of speech segments. The significant difference in speech duration may support the idea that the actual production of the sounds/phonological forms are affected in PCA patients. Importantly, long silent pauses, which are considered a significant indicator for LvPPA speech, were not found in this dataset of PCA patients.

In the stimuli set there were 8 short items (e.g., *You know how*) and 8 longer items (e.g., *Go ahead and do it if possible*). Analysis of the effect of sentence length on repetition showed that there were no significant length effects evident in patients or controls (*p* > 0.05). Patients and controls showed similar impaired accuracy and temporal characteristics in both and long and short sentences. It is likely that, although the longer sentences have a higher number of words/syllables, the sentences are still not complex embedded sentences that may evoke a length effect.

### 3.3 Working memory and attentional control assessment

PCA patients were significantly impaired on digit span backwards, Hayling Section 1 response initiation, Hayling Section 2 response suppression and Hayling overall score, compared to controls.

Multiple regression analyses revealed that performance on the Hayling sentence completion task predicted accuracy of sentence repetition. The overall model for the predictive value of attentional control measures was significant [*F*(5,17) = 5.668, *p* = 0.003, *R*^2^ = 0.625]. Hayling Section 2 response inhibition (*p* = 0.002), Hayling Section 2 errors (*p* = 0.014) and Hayling overall score (*p* = 0.011), were all significant predictors.

Regression analyses revealed no significant association between the Haying sentence completion task and LD or speech duration. There was no significant association between Digit span metrics and accuracy, LD or speech duration.

## 4 Discussion

Previous research has given no clear indication of whether sentence repetition is a common impairment in PCA. The present findings show that there is subtle impairment in sentence repetition in the majority of PCA patients. The current findings extend our understanding of the language profile in PCA by presenting converging evidence across several indices to suggest an interplay between multiple linguistic and cognitive processes in the breakdown of sentence repetition.

The literature on language impairments in PCA has implicated a phonological working memory deficit, similar but milder to that reported in LvPPA, and linked to the atrophy of the left temporoparietal cortex (Tetzloff et al., 2021), a key region suggested to subserve the phonological store (Paulesu et al., 1993). PCA patients showed overall reduced accuracy in sentence repetition. Hohlbaum et al. (2018) suggest that the words that have to be remembered in the sentence act as distractors, competing for resources in the phonological buffer. With reduced capacity due to impaired phonological processes, overall accuracy of repetition is compromised. Significantly higher LD scores suggest that PCA made significantly more errors compared to controls in repeating the original sentence, characterized by sound substitutions, omissions and additions, again suggesting a difficulty with phonological processes. LD is an extensively researched measure in the a peadiatric language impairment literature, and has been shown to have a strong correlation with phonological working memory (Riches, 2012).

Language impairment in PCA has also previously been attributed to impaired lexical-semantic retrieval (Crutch et al., 2013). The current findings suggest that, in sentence repetition at least, impaired semantic retrieval is not a predominant feature. There was no reduction in CIUs suggesting that the semantic content and informativeness of the sentence remained normal, with no evidence of the addition of verbal information that did not form part of the original stimulus. Since the lexical form was provided (as the task was a repetition task), it is not surprising the lexical retrieval for the target words was largely accurate.

Longer preparation time and longer utterance duration add further credence to the interplay of multiple linguistic and cognitive abilities in subtly impaired language in PCA. PCA patients took significantly longer to initiate their response, a not uncommon finding in neurological and aging populations and shown to reflect time involved in semantic, lexical and syntactic planning and phonological retrieval (Salis et al., 2018; DeDe and Salis, 2020; Salis et al., 2021). The longer utterance duration reflects time to continued linguistic planning and execution along with execution of the speech segments (Levelt, 2001), and indicated by three subcomponents: silent pauses, speech duration and dysfluency duration. Interestingly, silent pauses were comparable to controls. Prolonged silent pauses are a defining feature of logopenic speech (Wilson et al., 2010; Leyton and Hodges, 2013), reflecting impaired lexical selection and semantic processing within a sentence. PCA patients showed no significant difference in pauses compared to controls, in line with previous findings that lengthy pauses are not a common feature of PCA speech (Crutch et al., 2013).

Of note, speech duration was increased in PCA patients suggesting difficulties with the actual articulation of words. This is a surprising finding given there is no mention in the literature, to date, of difficulty with articulation or motor speech aspects in PCA. Indeed, this remains the case when taken together with dysfluency duration in which PCA patients were comparable to controls. If motor speech was impaired, it would be expected that dysfluency duration would also be longer in patients. Instead, the findings suggest that PCA have difficulties with producing the actual words of the stimuli, as opposed to a generalized motor speech issue, which can be linked to the influence of linguistic processes on speech movement (e.g., Strand and McNeil, 1996; Bose and van Lieshout, 2008). It is possible that PCA patients may be slowing down their speech as a strategy to correctly articulate meaningful words. This could be a conscious strategy or a function of impaired executive abilities that were evident in PCA, based on evidence for a concurrent relationship between the two faculties (Netelenbos et al., 2018).

Taken together, the findings suggest that though PCA is commonly considered to share an LvPPA-like profile of language impairment, there are key differences that point to the vulnerability of similar cognitive systems with subtle differences in clinical presentation. From a theoretical standpoint, the findings indicate the imbricated involvement of phonological, planning and attentional control processes in the subtle impairment of language in PCA. Impaired attentional control, i.e., the ability to focus on relevant information and resist environmental, cognitive or other distractions, to permit successful retention of memory units (Shipstead et al., 2014), in PCA patients aligns with theoretical accounts of repetition impairment in the literature.

The significant increase in preparation time and utterance duration suggests that PCA patients took longer to plan and then execute responses due to impaired focus and ability to limit irrelevant distractors. This may also underpin poorer performance on backward digit span compared to forward, as backward span demands greater attentional control due to the manipulation of the digits into reverse order. With the caveat of a small sample size in this study, potential modulation of sentence repetition by impaired attention control is further indicated by the association of Hayling sentence completion test and accuracy of sentence repetition. PCA patients were impaired on both response initiation and inhibition, but only response inhibition predicted accuracy of sentence repetition, lending further credence to the proposal that PCA patients were impaired in their ability to focus and limit distractors. There is a robust body of evidence highlighting the role of the posterior parietal cortex in supporting of attentional mechanisms (Corbetta, 1998; Cabeza et al., 2008; Hutchinson et al., 2009). We have previously shown that lateral and medial parietal cortical regions classically impaired in PCA, are critical components of the structural and functional networks implicated in attention and executive functions (Ahmed et al., 2018; Veldsman et al., 2019). It is possible that deficits in these complex systems impact language processing in PCA. It is important to acknowledge that the Hayling Sentence Completion task also requires lexical-semantic retrieval, shown to be subtly impaired in PCA. Thus we do not conclude that attentional control alone underpins poor sentence repetition in PCA, but is indicated as contributory. There is a dearth of evidence in this area and further studies exploring the nature of working memory, attentional control and language deficits and their neural underpinnings in PCA are warranted.

While PCA patients were impaired on digit span, there was no association with sentence repetition metrics, as expected. A potential relationship warrants further exploration in a larger sample. Another possible consideration could be that sentence repetition tasks tap into more than just phonological working memory. Repetition also reflects the integrity of the semantic and phonological long term store, mediated by attentional control. By contrast, digit span is a purer indication of phonological working memory with less access to long term memory needed to complete the task.

From a practical perspective, the present findings have a number of clinical implications. Detailed delineation of the language profile in PCA contributes to the improved characterization of the syndrome, and the relevance of secondary impairments that may follow from the salient neurodegeneration and primary cognitive impairments subserved by the posterior parietal cortex. Sentence repetition as a task is particularly useful in this endeavor given that expressive speech, in general, is relatively spared in PCA, and the restrictions imposed by the salient visual disorder in PCA that prevent accurate use of common visual tasks like picture naming and picture description tasks. It is a highly constrained task since the full content of the sentence is given, diminishing the influence of any word-finding difficulties. Repetition does not require post-interpretative processing as the meaning of the sentence does not need to be deciphered to complete the task, and so errors can be attributed to processes internal to sentence repetition. This ability of the task in identifying specific linguistic processes has been utilized for early detection and diagnosis of language impairments in a wide range of developmental language disorders, including establishing performance on sentence repetition as a diagnostic marker (Pawlowska, 2014; Rujas et al., 2021). Finally, delineating the presence and type of language deficits in PCA plays a critical role in patient management, guiding discussions on disease management strategies and development of tailored interventions (Burbaite et al., 2024).

There are a number of caveats to the findings. The sample of PCA patients was relatively small, in part due to the relativity rarity of PCA patients compared to other more common dementia syndromes. Still, the findings are in line with previous conclusions drawn about language deficits and modulating cognitive mechanisms in PCA, suggesting the present sample was representative of PCA. Future research should consider the use of additional, more nuanced sentence repetition stimuli such as the Litmus Sentence Repetition Task (Marinis and Armon-Lotem, 2015) to capture additional informative aspects of linguistics processing, such as morphosyntactic features, length, sentence types which stand to further refine understanding of language in PCA. Incorporation of a broader selection of neuropsychological measures would help to further delineate the role of attention and processing speed on sentence repetition capability in PCA, including measures of simple auditory processing and linguistic and non-linguistic processing speed. In addition, neuroimaging was not accessible for all patients and controls in this sample. Further study in a larger sample with detailed neuroimaging is certainly warranted to develop these findings.

To conclude, impaired sentence repetition is a common finding in the Alzheimer’s spectrum disorders, modulated by different cognitive systems. Sentence repetition is a defining feature of LvPPA due to phonological storage deficits, and a common finding in amnestic AD due to executive deficits. Here, we add preliminary evidence for impaired sentence repetition in PCA characterized by phonological and planning difficulties, underpinned by attentional control mechanisms and consistent with the defining temporoparietal degeneration.

## Data availability statement

The raw data supporting the conclusions of this article will be made available by the authors, without undue reservation.

## Ethics statement

The studies involving humans were approved by National Research Ethics Service South Central - Hampshire B and Oxford C. The studies were conducted in accordance with the local legislation and institutional requirements. The participants provided their written informed consent to participate in this study.

## Author contributions

SA: Conceptualization, Data curation, Formal analysis, Funding acquisition, Investigation, Methodology, Project administration, Resources, Software, Supervision, Validation, Visualization, Writing – original draft, Writing – review & editing. JC: Conceptualization, Data curation, Formal analysis, Investigation, Methodology, Software, Writing – review & editing. CB: Funding acquisition, Resources, Writing – review & editing. AB: Conceptualization, Data curation, Formal analysis, Methodology, Software, Supervision, Writing – review & editing.
